# Using Combination therapy to overcome diverse challenges of Immune Checkpoint Inhibitors treatment

**DOI:** 10.7150/ijbs.93697

**Published:** 2024-07-15

**Authors:** Racheli Birnboim-Perach, Itai Benhar

**Affiliations:** 1 Department of Molecular Microbiology and Biotechnology, The Shmunis School of Biomedicine and Cancer Research, The George S. Wise Faculty of Life Sciences, Tel-Aviv University, Tel-Aviv 69978, Israel.; 2 Cancer Biology Research Center, Tel Aviv University, Tel-Aviv 69978, Israel.; 3 The Tel Aviv University Center for Combatting Pandemics, Tel-Aviv University, Tel-Aviv 69978, Israel.

## Abstract

Immune checkpoint inhibitors (ICIs) have heralded a new era in immunotherapy, representing a pivotal breakthrough in cancer treatment. Their impact is profound, with ICIs standing as some of the most prescribed anticancer therapies today. Notably, their ability to induce long-term remission even after treatment cessation provides genuine hope for achieving durable cures. However, despite these strides, challenges persist in the landscape of oncology, including resistance phenomena, immune-related adverse events, and suboptimal response rates.

In response to these challenges, combination therapy emerges as a promising approach, poised to enhance treatment outcomes and address limitations inherent to single-agent ICI therapy. By synergistically targeting multiple pathways, combination therapy holds the potential to augment therapeutic efficacy while mitigating toxicity and impeding the emergence of resistance mechanisms. Understanding the intricacies underlying resistance development and adverse events is paramount in devising novel and refined combination strategies. A timeline showing FDA approvals of ICIs combination is shown in **Figure 1**.

This review aims to provide a comprehensive and up-to-date examples of different combined therapy strategies that can be used to overcome various challenges regarding ICI treatment. Through the exploration of innovative therapeutic combinations, we aim to provide clinicians and researchers with actionable knowledge to optimize patient outcomes and propel the field of immuno-oncology forward.

## Introduction

In cancer treatment, immunotherapy represents a strategic approach that harnesses various elements of the immune system to combat cancerous cells. This therapeutic paradigm involves the utilization of diverse pharmacological agents to target specific proteins found on the surface of either cancer cells or immune cells^1^,^2^. Central to this strategy are immune checkpoint inhibitors (ICIs), a subclass of immunotherapeutic agents designed to provoke an immune response against tumors by targeting key regulatory proteins. Among the most prominent ICIs are monoclonal antibodies that selectively bind to and inhibit immune checkpoint proteins, notably cytotoxic T lymphocyte-associated protein 4 (CTLA-4), programmed cell death 1 (PD-1) receptors, and its ligand PD-L1. These proteins are pivotal in modulating T cell activation and when targeted by ICIs, can potentiate an anti-tumor immune response^3^.

The approval of immune checkpoint modulators by the FDA in 2011 marked a significant breakthrough in immunotherapy. Since then, ICIs have catalyzed a paradigm shift in cancer treatment, emerging as cornerstone therapies across a spectrum of malignancies. Notably, anti-PD-1/PD-L1 antibodies have earned recognition as standard-of-care treatments for more than 50 cancer types^4^. Despite the remarkable advancements facilitated by ICIs in oncology, several significant challenges persist within the treatment landscape^5^. These challenges include primary and secondary resistance to therapy^6^, immune-related adverse events (irAEs)^2^, and suboptimal treatment responses, leading to the inability to achieve desired treatment outcomes. Recognizing the complexity of cancer and the multifaceted nature of therapeutic resistance, combination therapy (CT) has emerged as a promising approach to surmount these obstacles and enhance treatment efficacy. The rationale behind exploring multifaceted targeting strategies lies in the acknowledgment that common diseases, such as cancer, are inherently multifactorial and thus unlikely to be effectively controlled or contained by single-pathway interventions alone^7^. By concurrently targeting distinct biological pathways or mechanisms, CT holds the potential to synergistically augment therapeutic efficacy while alleviating adverse effects and slow down the emergence of drug resistance. This comprehensive approach aims to optimize treatment outcomes by leveraging the complementary actions of multiple agents.

In this article, we intend to conduct a critical review of the diverse strategies employed in combined therapy and to address the challenges associated with ICI treatment. By exploring various combination approaches, we aim to provide insights into novel therapeutic paradigms that hold promise in overcoming the complexities of immunotherapy resistance and enhancing patient responses to treatment.

### The Immune Checkpoint Inhibitors era - remarkable contributions alongside ongoing challenges

Immune checkpoints constitute a crucial regulatory mechanism in the immune system, comprising a network of receptors and ligands tasked with maintaining immune balance. Their primary function is to prevent excessive immune activation and protect normal cells from collateral damage during immune responses unrelated to pathology. Immune checkpoints play a crucial role in maintaining self-tolerance by dampening the activation of T cells, preventing harmful immune reactions from occurring at inappropriate times or locations. Regrettably, cancer cells may exploit these regulatory pathways to evade immune surveillance, hijacking immune checkpoint signaling to suppress anti-tumor immune responses. In response, monoclonal antibodies targeting immune checkpoints have emerged as potent therapeutic agents capable of reactivating the host immune system. By disrupting co-inhibitory signaling pathways, these antibodies unleash an anti-cancer immune response and provide new possibilities for cancer therapy^8^.

The approval of ipilimumab in 2011 marked a significant milestone in cancer treatment^9^. Targeting the CTLA-4 molecule, ipilimumab swiftly became a cornerstone therapy for advanced melanoma^9^. Subsequently, other agents blocking additional immune checkpoint pathways, such as the programmed cell death 1 (PD-1) and its ligand PD-L1, have also been approved^10^,^11^. PD-1 inhibitors include nivolumab, pembrolizumab, and cemiplimab, while PD-L1 inhibitors include atezolizumab, durvalumab, and avelumab^12^. ICIs have become the standard treatment for various types of solid tumors, including melanoma, non-small cell lung cancer, renal cell carcinoma, head and neck cancer, and gastrointestinal malignancies^13^-^15^. In hematological malignancies, nivolumab and pembrolizumab have been approved for treating relapsed and refractory classical Hodgkin lymphoma and primary mediastinal large B cell lymphoma^16^. These approvals underscore the expanding role of ICIs in reshaping therapeutic paradigms and offering hope to patients across diverse oncological settings.

One of the most significant breakthroughs achieved by ICIs is the potential for long-term remission even after discontinuation of treatment, instilling genuine hope for a curative approach in some patients^4^. However, this outcome remains out of reach for many, emphasizing the complexities of ICI therapy and the variability in patient responses. Despite being hailed as a transformative intervention in cancer care, ICIs are not universally effective, with a significant proportion of patients failing to derive substantial benefit^17^. Indeed, clinical data reveal that approximately 60-70% of patients with melanoma and lung cancer treated with ICIs do not exhibit a desirable response^18^. Within this cohort, some individuals demonstrate primary resistance to ICIs, while others acquire resistance following an initial positive response. Understanding the diverse mechanisms underpinning resistance is vital for enhancing treatment outcomes. To confront the challenge of resistance, novel combination strategies have emerged, involving the integration of ICIs with various agents such as radiotherapy, chemotherapy, targeted therapies, or next-generation immune modulators. These synergistic or additive approaches aim to amplify anti-tumor immune responses while circumventing mechanisms of resistance^19^.

Additionally, a subset of patients receiving ICI therapy may experience severe irAEs resembling autoimmune disorders, including autoimmune thyroiditis and inflammatory bowel diseases^4^,^6^. A large meta-analysis reported all-grade incidence of irAEs is about 83% with CTLA-4 inhibitors, 72% with PD-1 inhibitors, and 60% with PD-L1 inhibitors^20^. These adverse events stem from the fundamental action of ICIs in "releasing the brakes" on immune regulation^21^. irAEs can affect any organ system or tissue, however, the gastrointestinal tract, endocrine glands, skin, and liver are most commonly affected^22^. Although these side effects can be severe and even life-threatening, close monitoring and prompt intervention can enable the continuation of ICI therapy. Effective management often necessitates collaboration between oncologists and specialists across various medical disciplines^23^. Despite the potential severity of irAEs, emerging evidence suggests a paradoxical association between these adverse events and therapeutic efficacy, suggesting a complex interplay between treatment response and immune dysregulation^24^. In this review, we discuss some of the main combination strategies beyond ICI-ICI pairings, aiming to broaden therapeutic insights and enhance clinical outcomes in the pursuit of effective cancer immunotherapy^25^,^26^.

### Overcoming primary and secondary resistance

Resistance to ICIs can manifest as either primary or acquired, stemming from the complex and dynamic interplay among cancer cells, the tumor microenvironment, and the immune system^27^,^28^. The heterogeneous response rates observed across various tumor types highlight the complexity of ICI therapy, with only a subset of patients exhibiting favorable responses^29^. Broadly, patients' responses to ICI treatment can be categorized into three distinct populations: responders, characterized by a sustained positive response to the treatment; innate resistance, where patients show no initial response; and acquired resistance, whereby initial responders eventually lose responsiveness. To be able to understand how primary and secondary resistance evolve, a comprehensive grasp of the underlying mechanisms governing each stage of the ICI response model is crucial^30^. By understanding the different resistance mechanisms to immune checkpoint blockade, combination therapies can be developed to overcome resistance and treatment failure. When discussing how to overcome resistance to ICI, it's important to understand the terms "hot" and "cold" tumors. "Hot tumors" are characterized by high T-cell infiltration, increased interferon-γ (IFN-γ) signaling, high PD-L1 expression levels, and high tumor mutational burden (TMB)^31^. These types of tumors, such as melanoma and lung cancer, tend to be more responsive to ICIs^32^. In contrast, “cold tumors” are characterized by the lack of T-cell infiltration, low TMB, low major histocompatibility complex (MHC) class I expression, and low PD-L1 expression^31^. These types of tumors are much less responsive to ICIs treatment^33^. Combination therapy with ICIs has been suggested as a way to convert “cold tumors” into “hot tumors” which may make these tumors more responsive to ICI therapy. These treatment combinations are focused on promoting T-cell activation, T-cell expansion, and T-cell infiltration^34^.

It is generally accepted that the reasons for failure of ICI therapy can be divided into three main categories: the first category is failure in generating effective anti-tumor T cells, such failure can result from low levels of adequate neoantigens and deficiencies in neoantigen presentation^35^. It has been shown that high TMB and elevated neoantigen expression, have an important role in antitumor immune response^36^,^37^. The second category of ICI therapy failure is insufficient function of tumor-specific T cells, this category is attributable to both intrinsic and extrinsic factors within the tumor microenvironment (TME)^38^. The TME, comprising a diverse array of immune cells, fibroblasts, and signaling molecules, harbors immune-suppressive elements such as regulatory T cells and inhibitory cytokines that thwart the anti-tumor immune response^39^,^40^. Lastly, the third category is impaired formation of effector memory T cells that can arise from profound T cell exhaustion or epigenetic alterations within T cells. These impairments compromise the generation of robust, long-lasting anti-tumor immune responses critical for sustained treatment efficacy^41^,^42^. By delineating these resistance mechanisms, novel therapeutic approaches can be tailored to counteract treatment resistance and enhance patient outcomes. Addressing the multifaceted challenges posed by ICI therapy demands a comprehensive understanding of the intricate immune landscape and the dynamic interactions within the tumor microenvironment.

Several combination strategies involving ICIs are undergoing clinical evaluation, with several already approved for clinical use. A summary of the combination strategies mentioned in this section are listed in **Table 1**. One particularly effective combination strategy involves pairing ICIs with chemotherapy. Chemotherapy can interact with different components of the immune system, enhancing immunogenicity while dampening immunosuppressive features within the TME. Certain chemotherapeutic agents augment tumor infiltration and increase the activity of effector cells such as cytotoxic T lymphocytes, dendritic cells, and natural killer cells. Additionally, other chemotherapies have the potential to deplete immune-suppressive cell populations like regulatory T cells. For instance, the combination of pembrolizumab with pemetrexed and platinum chemotherapy has demonstrated significant improvements in overall survival (OS) and progression-free survival (PFS) in metastatic non**-**squamous non-small cell lung cancer (NSCLC) 43,44. Platinum compounds have been shown to recruit dendritic cells, induce their maturation, and enhance antigen presentation within the TME^45^. Similarly, combining pembrolizumab with carboplatin and paclitaxel or nab-paclitaxel has yielded notable OS and PFS benefits in metastatic squamous NSCLC, with nab-paclitaxel selectively depleting myeloid-derived suppressor cells^44^.

An interesting innovation in the field of chemotherapy are Antibody-Drug Conjugates (ADCs). ADCs are an advanced class of potent anti-cancer compounds that unlike conventional chemotherapies, combine the potency of anti-cancer drugs with the specificity of mAbs^46^,^47^. Several preclinical and clinical trials support combination therapy with ADCs and ICIs for treating NSCLC. Initial results from the TROPION-Lung02 trial showed encouraging efficacy and safety when combining datopotamab deruxtecan (an anti-trophoblast cell surface protein 2 (TROP2) mAb, conjugated to a potent DNA topoisomerase I inhibitor) and pembrolizumab with or without platinum chemotherapy^48^,^49^.

A class of small-molecule-drugs with a unique mechanism of action are the epigenetic agents (epidrugs). These are drugs that modulate epigenetic modifications such as DNA methylation (such as DNMT inhibitors), histone modifications (such as HDAC inhibitors) and the involvement of ncRNAs, which are believed to comprise the primary regulators of pathophysiological progression. The pivotal roles of epigenetic mechanisms in cancer initiation and progression, as well as in cell fate and functioning of the immune system, led to studies of epidrugs as anti-cancer drugs. In several such studies, combinations of epidrugs with ICIs have been studies as well. To date, combinations of epidrugs with ICIs were evaluated in small patient groups in phase I or II clinical trials, where patients suffering from hematological malignancies and from various carcinomas were treated. Such studies provided promising evidence that combinatorial strategies employing epigenetic drugs along with ICIs provide a novel option for cancer treatment. Further investigations are required to assess the clinical efficacy of combining epigenetic drugs with ICIs. In this way, it will be possible to develop new and improved rationale-based combinational strategies that will significantly enhance the practice of cancer immunotherapy in the future^50^.

Another potent combination approach that can be used to overcome resistance involves the combination of ICIs with targeted agents such as small molecule kinase inhibitors. For instance, the combination of vemurafenib (a BRAF inhibitor) and cobimetinib (a MEK1 inhibitor) with atezolizumab (a PD-L1 inhibitory mAb) has shown improved PFS in patients with unresectable or metastatic melanoma^51^. Vemurafenib has been observed to enhance antigen processing and increase the expression of major MHC molecules^52^. An additional strategy is to combine ICIs with antiangiogenic drugs targeting vascular endothelial growth factor (VEGF) or its receptor (VEGFR). VEGF secretion within the TME blocks T cell development and infiltration while promoting the proliferation of immune-suppressive cells^53^. This combination therapy has shown efficacy in numerous clinical trials, leading to FDA approvals for several combinations. The administration of antiangiogenic drugs can reverse immune suppression by increasing intra-tumoral effector cells, decreasing PD-L1 expression, and reducing infiltrating myeloid-derived suppressor cells (MDSCs) and regulatory T cells^54^,^55^. For instance, combination of pembrolizumab with the multiple receptor tyrosine kinase inhibitor lenvatinib has been evaluated in the treatment of renal carcinoma in patients with favorable risk or intermediate/poor risk and in endometrial carcinoma. The authors suggested that the use of non-chemotherapy containing regimens may spare patients from extended durations of myelosuppression and reduce the risk of infection. Additionally, pembrolizumab with lenvatinib demonstrates efficacy as a first line treatment in clear cell renal carcinoma, second line in endometrial carcinoma, and several potential uses on the horizon^56^.

Furthermore, the combination of ICIs with anti-CD20 monoclonal antibodies like rituximab has demonstrated promise in overcoming resistance mechanisms, particularly in melanoma. Tumor-associated B cells have been implicated in drug resistance and the secretion of pro-tumorigenic factors such as IGF-1 secretion^57^, making them viable targets for therapeutic intervention. Data from case series showed median survival exceeding 1 year in patients with multi-treated metastatic melanoma receiving rituximab^58^.

### Overcoming severe irAEs and toxicity

In the context of irAEs, the ICI treatment can be described as a double-edged sword. Releasing the physiological brakes of the immune system to induce a strong anti-tumor immune response is the treatment's mechanism of action. However, the same strong immune response can often result in off-target effects and lead to immune-mediated adverse events, which can resemble autoimmune disorders^59^. The incidence of irAEs spans a wide spectrum, ranging from 10% to 90%, with severe irAEs (grade 3 or higher) affecting 2.5% to 18% of patients receiving ICIs^60^. Typically, irAEs manifest within the initial weeks of treatment initiation, although they can emerge at any time point, including after treatment cessation. Alarmingly, up to 19% of patients in ICI clinical trials have had to discontinue treatment due to irAEs^61^-^63^. Notably, the incidence of irAEs varies among different ICIs, with anti-CTLA-4 inhibitors associated with the highest occurrence^64^.

Predictably, irAEs differ markedly from adverse events linked to conventional cytotoxic or targeted therapies, presenting clinicians with a distinct set of clinical challenges. While dermatitis and thyroiditis are common irAEs, more serious manifestations such as pneumonitis, colitis, and hepatitis can occur, albeit less frequently^23^. Importantly, patients with pre-existing autoimmune diseases pose a unique management challenge, as they are at heightened risk of experiencing disease flares while undergoing ICI therapy^65^. Treating patients with pre-existing autoimmune diseases can be challenging, however, evidence is accumulating regarding ICI use in these patients, with reassuring safety data reported in several case reports and in several retrospective series^66^-^68^. Managing the treatment of patients with irAEs, whether it is "de novo” induced irAEs or a flare of pre-existing autoimmune disease, requires efficient and multidisciplinary monitoring.

Despite the exact pathophysiology of irAEs remaining elusive, several mechanisms are proposed, including the activation of self-reactive T cells^69^, antigenic overlap between cancer cells and affected tissues^70^, direct toxicity to organs expressing immune checkpoint proteins, dysregulation of inflammatory cytokines and chemokines^71^ and decreased levels of regulatory T cells^72^. Several guidelines on the management of irAEs have been published^73^-^75^. These guidelines provide key recommendations for managing immunotherapy-related toxicity and include assessment and treatment algorithms according to the grade of toxicity. As a first line approach, irAEs can be treated with Glucocorticoids such as prednisolone or parenteral methylprednisolone^76^. However, severe, and life-threatening irAEs are more challenging to diagnose and treat. Since there is a lack of prospective trials on drug immunosuppression in the setting of severe irAEs, information on how to manage such irAEs is collected based on a small series of studies and case reports. It has been suggested that novel biological agents targeting key inflammatory components can be used based on the immunopathological patterns of each patient^77^. A summary of the combination strategies mentioned in this section are listed in **Table 2**.

For instance, colitis induced by ICIs can be effectively managed with a single dose of infliximab, a chimeric monoclonal antibody targeting tumor necrosis factor alpha (TNFα). This approach has demonstrated efficacy in treating corticosteroid-refractory ICI-related colitis^78^. Alternative anti-TNFα agents such as etanercept, adalimumab, certolizumab, and golimumab may also be considered. In severe cases of ICI-induced polyarthritis, adalimumab has shown success in alleviating joint inflammation^79^. Another therapeutic approach involves blockade of interleukin-1 (IL-1) using agents like anakinra or canakinumab, which have shown promise in managing various irAEs including myasthenia gravis, encephalitis, severe arthritis, and psoriasis^80^. IL-1 inhibition is particularly beneficial in ICI-related irAEs due to its prominent role in acute inflammation, with no negative impact on cancer response^81^. Similarly, interleukin-6 (IL-6) blockade with tocilizumab, an IL-6 receptor inhibitory monoclonal antibody, holds potential for managing steroid-refractory irAEs. Aside from the anti-inflammatory benefits, IL-6 is also known to promote cancer development and metastasis. Thus, by targeting IL-6 while treating with ICI, a synergistic effect could be achieved^82^,^83^. Possible indications for anti-IL-6 therapy include severe irAEs such as severe arthritis, uveitis, Graves' orbitopathy, myocarditis, large-vessel vasculitis, severe pneumonitis, and myasthenia gravis^84^-^86^.

While the pathogenic role of T cells in irAEs is well-established, emerging evidence suggests a contribution from B cells as well^87^,^88^. ICI-related encephalitis, an uncommon yet severe manifestation of irAEs, is associated with the presence of anti-neural autoantibodies^89^. Rituximab, an anti-CD20 monoclonal antibody, has shown impressive neurological improvement in patients with ICI-related encephalitis. In these cases, the patients were first treated with corticosteroids and intravenous immunoglobulins (IVIG) without success^90^,^91^. IVIG, derived from pooled immunoglobulins of healthy donors, serves as a versatile therapeutic option for various immunodeficiency states and inflammatory conditions, including ICI-related irAEs. Standard IVIG protocols have demonstrated impressive efficacy in managing irAEs^92^. IVIG can be used in cases of immune thrombocytopenia which is a rare and life-threatening form of irAE that are corticosteroids-refractory (approximately 25% of patients)^93^. The judicious selection of immunomodulatory agents tailored to the specific irAE profile is paramount in achieving optimal outcomes while minimizing treatment-related toxicity. As our understanding of irAEs continues to evolve, personalized therapeutic approaches will play an increasingly pivotal role in navigating the complexities of ICIs therapy.

### Improving the treatment outcomes to ICI and increasing the indication range

The emergence of ICIs has heralded a paradigm shift in cancer immunotherapy, yet the quest for novel strategies to enhance anti-tumor responses and amplify treatment success continues. Among these strategies, combination therapy stands out, with extensive research endeavors aimed at identifying synergistic or additive regimens to augment ICI efficacy and expand its indications. One promising strategy involves combining ICIs with radiotherapy, a standard treatment modality for over 50% of patients suffering from tumors^94^. In this context, radiotherapy acts synergistically with ICIs by enhancing the expression of leukocyte adhesion molecules on endothelial cells and promoting the secretion of chemokines that attract CD8^+^ T cells^32^,^95^. Moreover, the combination of radiotherapy with targeting ICIs could provoke cytotoxic T cells-mediated anti-tumor immunity by inducing somatic mutations that can generate neoantigens, and further augmenting immune responses^96^. Clinical studies have demonstrated the therapeutic benefits of combining ICIs with radiotherapy in melanoma, NSCLC, renal cell carcinoma (RCC), and ongoing studies explore its applicability across diverse malignancies^97^,^98^. Recently, the combination of ICIs with radiotherapy was evaluated in hepatocellular carcinoma (HCC). That study demonstrated the advantage of combining ICI with radiotherapy compared to the combination of ICIs and antiangiogenic therapy, the inclusion of radiotherapy improved the disease control rate and survival outcomes in patients with advanced-stage HCC. The safety profile of this triple therapy was satisfactory^99^.

Another promising approach involves leveraging targeted small molecule drugs, which have become mainstream cancer therapies by targeting various cellular pathways including kinases, epigenetic regulators, DNA repair enzymes, and proteasomes^100^. Several studies have demonstrated the synergistic potential of these agents with ICIs to enhance treatment outcomes. Notably, the combination of ICIs with targeted therapy drugs inhibiting rapidly accelerated fibrosarcoma (RAF) and mitogen-activated protein kinase kinases (MEK) is the most widely studied combination of small molecule drugs and ICIs. In melanoma patients harboring the BRAF V600E mutation, combining BRAF and MEK inhibitors significantly enhances treatment efficacy. BRAF inhibition reduces anti-inflammatory cytokine levels that suppress tumor-infiltrating lymphocytes (TILs). Moreover, PD-L1 is upregulated in patients resistant to BRAF inhibitors^101^,^102^. This synergistic interplay between ICIs and BRAF inhibitors has yielded impressive outcomes, exemplified by the combination of dabrafenib (a BRAF inhibitor), trametinib (a MEK inhibitor), and spartalizumab (an anti-PD-1 mAb), which demonstrated a remarkable 100% response rate and reduced relapse compared to BRAF-MEK inhibitor monotherapy in melanoma patients^103^-^105^.

Exploring multiple targeting strategies for ICIs serves another crucial purpose: expanding the spectrum of indications to encompass a wider array of cancer types. Initially, breast cancer was not among the cancer subtypes investigated with immune checkpoint inhibitor. Early clinical trials investigating PD-1/PD-L1 monoclonal antibodies as monotherapy for triple-negative breast cancer (TNBC) yielded modest to negligible clinical benefits^106^-^108^. However, in 2019, atezolizumab received accelerated approval for first-line treatment of metastatic TNBC in combination with nanoparticle albumin-bound (nab)-paclitaxel^107^.

Despite this approval, metastatic TNBC is still one of the most challenging cancer types to treat, prompting ongoing efforts to identify novel combinational regimens to enhance response rates. One of the options that are under clinical development is targeting the human epidermal growth factor receptor 2 (HER-2) oncogene. HER-2-targeted therapy such as trastuzumab, have revolutionized the prognosis for patients with HER2-positive breast cancer^109^. However, a subset of patients develops resistance to trastuzumab, characterized by upregulated PD-1/PD-L1 expression within the TME^110^. Consequently, clinical trials evaluating the combination of trastuzumab with pembrolizumab have been undertaken in patients with advanced or metastatic HER2-positive breast cancer refractory to trastuzumab. Preliminary data from these trials have demonstrated promising therapeutic outcomes, including a 15% response rate in PD-L1-positive tumors and a 12-month progression-free survival rate of 13%^111^. Building upon these advancements, in 2021, the FDA granted accelerated approval to pembrolizumab in combination with trastuzumab and fluoropyrimidine-and platinum-containing chemotherapy for first-line treatment of patients with locally advanced, unresectable, or metastatic HER2-positive gastric adenocarcinoma^112^. A summary of the combination strategies mentioned in this section are listed in **Table 3**.

## Conclusion

Undoubtedly, ICIs have become a cornerstone in the management of malignancy, affording many cancer patients improved outcomes and extended survival. Yet, primary and secondary resistance, along with the occurrence of severe side effects and low response rates, underlines the ongoing challenges associated with ICI therapy. In response, clinicians and researchers are actively exploring various ways to enhance the efficacy of ICIs, with combination therapy standing out as a promising approach.

**Figure 2:**
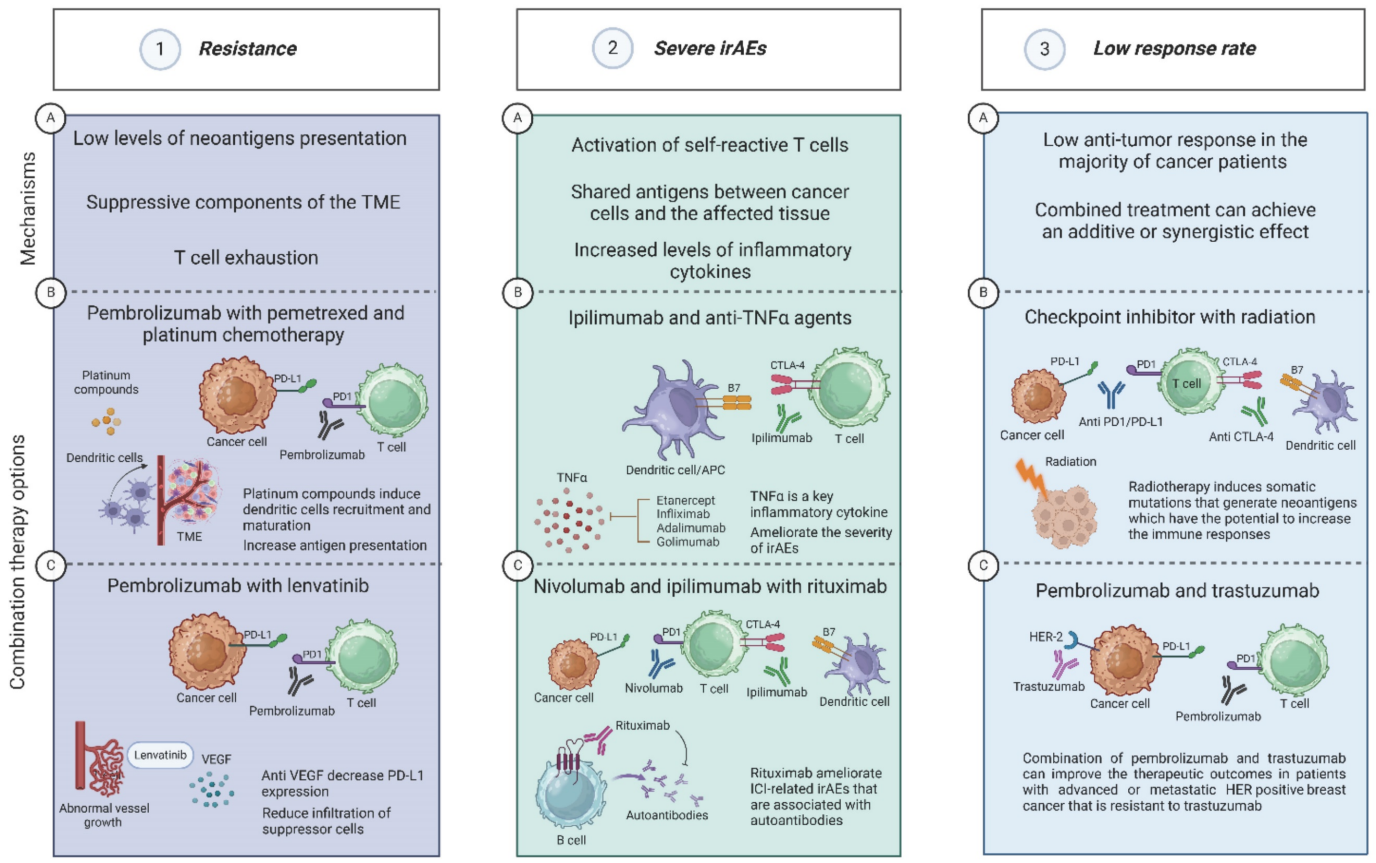
** Different drug combinations with ICI can be used for different purposes. Panel 1. Resistance**. **1A.** The main mechanisms that can lead to the development of ICI-resistance are low levels of neoantigens presentation, suppressive components in the TME, and T cell exhaustion^35^,^38^,^41^. **1B**. The combination of pembrolizumab with pemetrexed and platinum compounds can be used to overcome ICI-resistance. Platinum compounds induce dendritic cells recruitment and maturation and increase antigen presentation^43^-^45^. **1C.** Pembrolizumab and anti VEGF such as Lenvatinib. Lenvatinib can decrease the levels of PD-L1 expression and reduce the infiltration of suppressor cells such as myeloid-derived suppressor cells (MDSCs) and regulatory T cells^54^,^55^. **Panel 2. Severe irAEs. 2A.** The main mechanisms that can lead to the development of irAEs are activation of self-reactive T cells, shared antigens between cancer cells, and increased production of inflammatory cytokines and chemokines^69^-^71^. **2B.** The combination of ipilimumab and anti-TNFα agent such as infliximab was shown to be effective for treating corticosteroid-refractory ICI-related colitis^78^. In addition, adalimumab was successful in ameliorating the severity of ICI-related joints inflammation^79^. **2C.** AntI-CD20 agents such as rituximab were effective in ameliorating ICI-related irAEs that are associated with the detection of autoantibodies such as encephalitis^89^. **Panel 3. Low response rate. 3A.** There is an ongoing effort to find new strategies to enhance the anti-tumor response since most cancer patients will not achieve the treatment goals^17^. One of these strategies is to gain an additive or synergistic effect by using combination therapy. **3B.** Combining ICIs with radiotherapy has the potential to improve the treatment outcome. Following radiotherapy, some patients may express somatic mutations that generate neoantigens which have the potential to increase the immune responses^97^,^98^. **3C.** Another significant motivation to explore new CT with ICIs is to increase the range of indications for additional types of cancer, such TNBC. The combination of trastuzumab (an anti-HER-2 mAb) with pembrolizumab has been evaluated in clinical trials in patients with advanced or metastatic HER positive breast cancer that is resistant to trastuzumab. The data gathered from this trial showed promising therapeutic outcomes including 15% response rates in PD-L1-positive tumors with 13% 12-month PFS^111^. Created with BioRender.com.

The combination of ICIs with other drugs can significantly contribute to achieving the treatment goals, whether the goal is to overcome resistance, reduce serious and life-threatening side effects, or improve the overall response to the treatment. The judicious selection and integration of appropriate combination therapies tailored to each patient's unique circumstances are vital. In this review, we discussed how different combination therapies can be used for different purposes depending upon the specific drugs utilized. Furthermore, we elucidate the distinct mechanisms of action underlying each therapeutic combination, providing valuable insights into their rationale and potential clinical utility. A summary of the motivation rationales to combine ICIs with other drugs, and some examples of such combinations are shown in **Figure 2**.

As for future prospects of the field, it is important to note that some combination therapies designed to enhance treatment response or reduce resistance may inadvertently increase the risk of irAEs. Thus, a comprehensive understanding of the underlying mechanisms implicated in each treatment challenge is and will remain crucial for making informed and rational decisions regarding the selection of appropriate combination therapies that maximize patient benefit while minimizing risks. Moving forward, artificial intelligence (AI) may have the potential to open new possibilities in the field of immunotherapy. Machine learning methods have been proven as powerful tools to predict potential anti-cancer synergistic drug combinations, especially as the availability of large datasets has grown^113^. Continued research efforts and clinical trials are required to identify novel and refined combination strategies tailored to specific pathological conditions.

## Author contributions

All authors made a significant contribution to this review article, whether that is in the conception, acquisition of data, analysis and interpretation, or in all these areas; took part in drafting, revising and critically reviewing the article. All authors gave final approval of the version to be published and agree to be accountable for all aspects of the work. All authors have read and agreed to the published version of the manuscript.

## Figures and Tables

**Figure 1:**
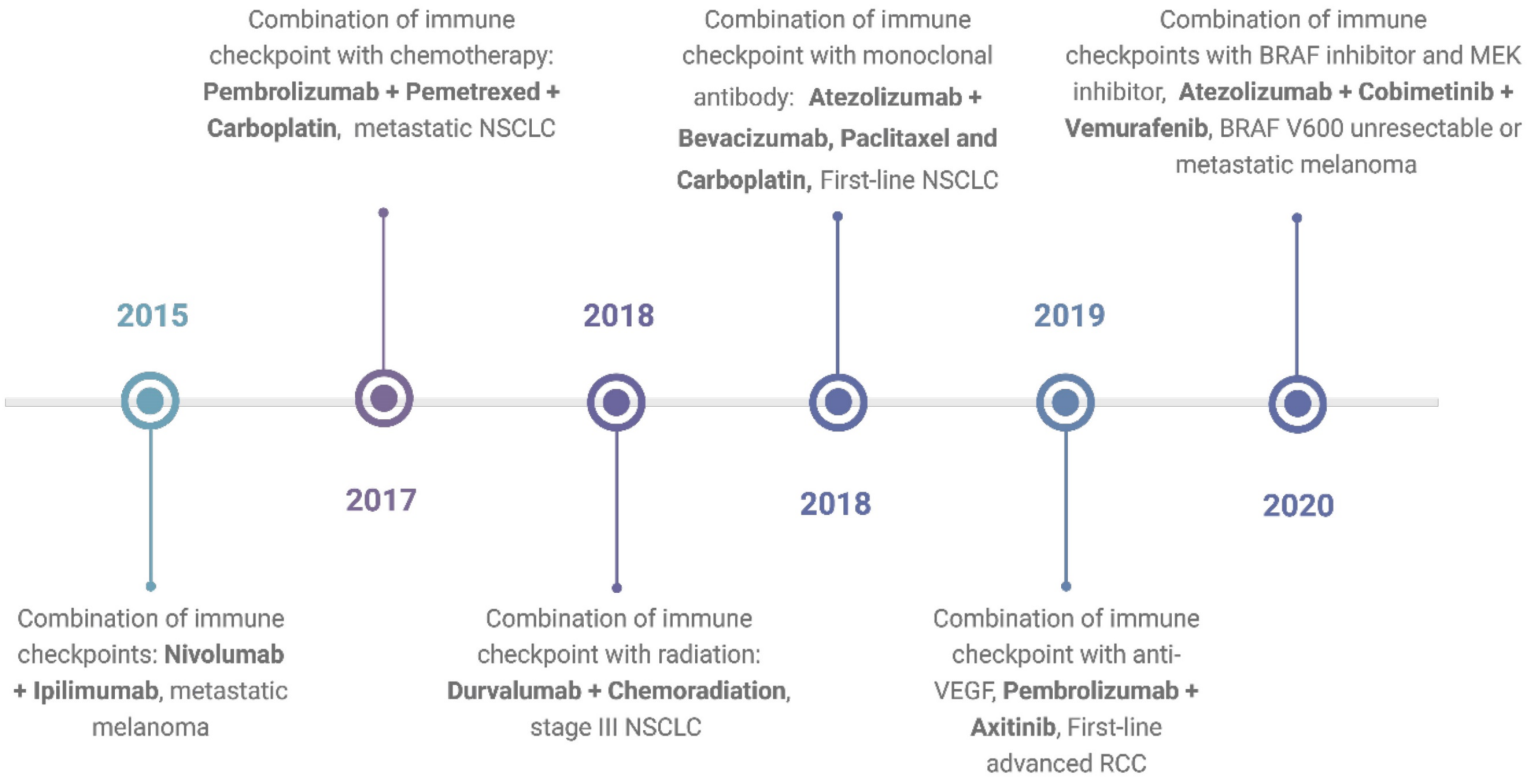
**Timeline of FDA-approved combination immunotherapies as of April 2024.** The timeline shows the first times the FDA approved a certain type of combination and the indication for which the combination was approved. The data is based on the FDA database. https://www.fda.gov/drugs/resources-information-approved-drugs/oncology-cancer-hematologic-malignancies-approval-notifications. Created with BioRender.com.

**Table 1:** Overcoming primary and secondary resistance:

Drug combination	Target	Mechanism/Rational	Outcomes	Indications	Reference
**Pembrolizumab** **Pemetrexed-Platinum chemotherapy**	PD-1 Key proteins that are abundant in cancer cells	Platinum compounds recruit dendritic cells, induce their maturation, and enhance antigen presentation within the TME	Improved OS and PFS compared with placebo plus pemetrexed-platinum	NSCLC	43, 44
**Pembrolizumab** **carboplatin and paclitaxel/nab-paclitaxel**	PD-1 DNA, Tubulin beta-1 chain	carboplatin and paclitaxel/nab-paclitaxel can lead to depletion of myeloid-derived suppressor cells	Significantly improved OS and PFS in comparison to chemotherapy alone	NSCLC	44
**Pembrolizumab** **Datopotamab deruxtecan**	PD-1 Trophoblast cell surface protein 2	ADCs combine the potency of strong chemotherapy drugs with the specificity of mAbs	Phase Ib trial (TROPION-Lung02) showed promising clinical activity in patients with advanced or metastatic NSCLC	NSCLC	48, 49
**Atezolizumab** **Vemurafenib with Cobimetinib**	PD-L1 BRAF MEK1	Vemurafenib can enhance antigen processing and increase the expression of major MHC molecules	Improved PFS	Unresectable or metastatic melanoma	51, 52
**Pembrolizumab** **Lenvatinib**	PD-1 VEGFR	Antiangiogenic drugs can increase intra-tumoral effector cells, decrease PDL1 expression, and reduce infiltration of MDSCs and regulatory T cells	Increase in PFS compared to sunitinib alone	Renal cell carcinoma, Endometrial carcinoma	53, 54, 55, 56
**Ipilimumab** **Rituximab**	CTLA-4 CD20	Depletion of tumor-associated B cells that have been implicated in drug resistance and the secretion of pro-tumorigenic factors	Median survival exceeding 1 year in patients with multi-treated metastatic melanoma	Metastatic melanoma	57

**Abbreviations**: PD-1 programmed cell death protein 1, PD-L1 programmed death-ligand 1, CTLA-4 cytotoxic-T-lymphocyte-associated protein 4, TME tumor microenvironment, MDSC myeloid-derived suppressor cells, OS overall survival, PFS progression-free survival, MHC major histocompatibility complex, NSCLC non-small cell lung cancer.

**Table 2:** Overcoming severe irAEs and toxicity:

Drug combination	Target	Mechanism/Rational	Outcomes	Indications	Reference
**ICI** **anti-TNFα agents**	PD-1, PD-L1, CTLA-4 TNFα	TNFα is a potent pro-inflammatory cytokine that play an important role in the pathogenesis of chronic inflammatory diseases, such as colitis and arthritis.	Success in managing the inflammation and alleviating symptoms	Corticosteroid-refractory ICI-induced polyarthritis and ICI-induced colitis	78, 79
**ICI** **Anakinra or Canakinumab**	PD-1, PD-L1, CTLA-4 IL-1	IL-1 inhibition is beneficial in ICI-related irAEs due to its prominent role in acute inflammation, with no negative impact on cancer response.	Alleviation of disease symptoms	Myasthenia gravis, encephalitis, severe arthritis, and psoriasis	80, 81
**ICI** **Tocilizumab**	PD-1, PD-L1, CTLA-4 IL-6	Aside from the anti-inflammatory benefits, IL-6 is also known to promote cancer development and metastasis. Thus, by targeting IL-6 while treating with ICI, a synergistic effect could be achieved	Alleviation of disease symptoms	Severe arthritis, uveitis, graves' orbitopathy, myocarditis, large-vessel vasculitis, severe pneumonitis, and myasthenia gravis	82, 83, 84, 85, 86
**ICI** **Rituximab**	PD-1, PD-L1, CTLA-4 CD20	ICI-related encephalitis is associated with the presence of anti-neural autoantibodies	Impressive neurological improvement in patients with ICI-related encephalitis	ICI-related encephalitis	87, 88, 89, 90, 91
**ICI** **IVIG**	PD-1, PD-L1, CTLA-4 Various components of the immune system	Neutralization of pathogenic autoantibodies, prevention of B-cells proliferation, suppression of pro-inflammatory cytokines production	Alleviation of disease symptoms	Pre-existing paraneoplastic neuromuscular diseases in cancer patients, corticosteroids-refractory immune thrombocytopenia	92, 93

**Abbreviations**: ICI immune checkpoint inhibitor, PD-1 programmed cell death protein 1, PD-L1 programmed death-ligand 1, CTLA-4 cytotoxic-T-lymphocyte-associated protein 4, TNFɑ tumor necrosis factor alpha, IL-1 Interleukin-1, IL-6 Interleukin-6, IVIG intravenous immunoglobulin.

**Table 3:** Improving treatment outcomes to ICI and increasing the indication range

Drug combination	Target	Mechanism/Rational	Outcomes	Indications	Reference
**ICI** **Radiotherapy**	PD-1, PD-L1, CTLA-4 DNA	Induction of somatic mutations that can generate neoantigens, promotion of chemokines secretion that attract CD8+ T cells	Improved disease control and survival outcomes	NSCLC, RCC, HCC	32, 95, 96, 97, 98, 99
**Spartalizumab** **Dabrafenib with Trametinib**	PD-1 BRAF MEK1	BRAF inhibition reduces anti-inflammatory cytokine levels that suppress tumor-infiltrating lymphocytes (TILs)	High response rate and reduced relapse compared to BRAF-MEK inhibitor monotherapy	Melanoma	100, 101, 102, 103-105
**Pembrolizumab** **Trastuzumab**	PD-1 HER-2	Resistance to trastuzumab is characterized by upregulated PD-1/PD-L1 expression, thus, combination of trastuzumab with pembrolizumab can be beneficial	15% response rate in PD-L1-positive tumors and a 12-month PFS rate of 13%	Metastatic HER2-positive breast cancer, HER2-positive gastric adenocarcinoma	109, 110, 111, 112

**Abbreviations**: ICI immune checkpoint inhibitor, PD-1 programmed cell death protein 1, PD-L1 programmed death-ligand 1, CTLA-4 cytotoxic-T-lymphocyte-associated protein 4, NSCLC non small cell lung cancer, RCC renal cell carcinoma, HCC hepatocellular carcinoma, HER-2 human epidermal growth factor receptor 2, PFS progression-free survival.
